# Nicotinic Cholinergic System and COVID-19: In Silico Identification of an Interaction between SARS-CoV-2 and Nicotinic Receptors with Potential Therapeutic Targeting Implications

**DOI:** 10.3390/ijms21165807

**Published:** 2020-08-13

**Authors:** Konstantinos Farsalinos, Elias Eliopoulos, Demetres D. Leonidas, Georgios E. Papadopoulos, Socrates Tzartos, Konstantinos Poulas

**Affiliations:** 1 Laboratory of Molecular Biology and Immunology, Department of Pharmacy, University of Patras, Panepistimiopolis, 26500 Rio-Patras, Greece; kfarsalinos@gmail.com (K.F.); stzartos@gmail.com (S.T.); 2 Laboratory of Genetics, Department of Biotechnology, Agricultural University of Athens, Iera Odos 75, 11855 Athens, Greece; eliop@aua.gr; 3 Department of Biochemistry and Biotechnology, University of Thessaly, Biopolis, 41500 Larissa, Greece; ddleonidas@bio.uth.gr (D.D.L.); geopap@bio.uth.gr (G.E.P.)

**Keywords:** COVID-19, SARS-CoV-2, smoking, nicotine, nicotinic cholinergic system, inflammation, acetylcholine receptors

## Abstract

While SARS-CoV-2 uses angiotensin converting enzyme 2 (ACE2) as the receptor for cell entry, it is important to examine other potential interactions between the virus and other cell receptors. Based on the clinical observation of low prevalence of smoking among hospitalized COVID-19 patients, we examined and identified a “toxin-like” amino acid (aa) sequence in the Receptor Binding Domain of the Spike Glycoprotein of SARS-CoV-2 (aa 375–390), which is homologous to a sequence of the Neurotoxin homolog NL1, one of the many snake venom toxins that are known to interact with nicotinic acetylcholine receptors (nAChRs). We present the 3D structural location of this “toxin-like” sequence on the Spike Glycoprotein and the superposition of the modelled structure of the Neurotoxin homolog NL1 and the SARS-CoV-2 Spike Glycoprotein. We also performed computational molecular modelling and docking experiments using 3D structures of the SARS-CoV-2 Spike Glycoprotein and the extracellular domain of the nAChR α9 subunit. We identified a main interaction between the aa 381–386 of the SARS-CoV-2 Spike Glycoprotein and the aa 189–192 of the extracellular domain of the nAChR α9 subunit, a region which forms the core of the “toxin-binding site” of the nAChRs. The mode of interaction is very similar to the interaction between the α9 nAChR and α-bungarotoxin. A similar interaction was observed between the pentameric α7 AChR chimera and SARS-CoV-2 Spike Glycoprotein. The findings raise the possibility that SARS-CoV-2 may interact with nAChRs, supporting the hypothesis of dysregulation of the nicotinic cholinergic system being implicated in the pathophysiology of COVID-19. Nicotine and other nicotinic cholinergic agonists may protect nAChRs and thus have therapeutic value in COVID-19 patients.

## 1. Introduction

As the global pandemic of corona virus disease 2019 (COVID-19) was spreading, it was recognized early that the virus (SARS-COV-2) uses the angiotensin converting enzyme 2 (ACE2) as a receptor for cell entry [1]. The 3-D structure and the function of SARS-CoV-2 Spike Glycoprotein has been published [2] and the structure of the complex of SARS-CoV-2 Spike Glycoprotein with the ACE2 has been solved by cryo-EM experiments [3]. This has been the focus of the global research community, examining the interactions between disease conditions or medications and ACE2 expression [4,5]. Among the risk factors that have been examined is smoking, considering that it is a major risk factor for respiratory infections [6]. Until recently, smoking was associated with the down-regulation of ACE2 [7]. However, recent studies show that it may cause ACE2 up-regulation, and this may propagate viral spread and disease severity [8]. However, the association between smoking (and nicotine) and COVID-19 appears to be much more complex. Consistent clinical observations from retrospective cases series of COVID-19 patients have shown a low prevalence of smoking among hospitalized COVID-19 patients [9].

In April 2020, we hypothesized for the first time that the nicotinic cholinergic system (NCS) may be implicated in the pathophysiology of severe COVID-19, and we recently expanded our research on this hypothesis [9,10]. Immune dysregulation and cytokine storm appear to play a major role in the pathophysiology of severe COVID-19 [11]. The “cholinergic anti-inflammatory pathway” is an important regulator of the inflammatory response [12]. Its effects are mediated mainly by the vagus nerve and by α7 nicotinic acetylcholine receptors (nAChRs). The highest concentration of α7 nAChRs is observed in the hippocampus, the thalamic reticular nucleus, as well as the lateral and medial geniculate nuclei. On the cellular level, they are expressed both pre- and postsynaptically [13]. Presynaptically, they are positioned to allow Ca^2+^ influx into the synaptic bouton, and in this way, they play a major role in modulating the release of many neurotransmitters. Alpha7 nAChRs are also observed on macrophages and other immune cells and have been implicated in preventing sepsis and acute respiratory distress syndrome (ARDS) in animal models [13,14]. Alpha7 nAChRs are expressed, also, in human bronchial epithelial and endothelial cells [15], which are the major targets of SARS-CoV-2. Dysfunction of the NCS could explain other clinical manifestations of COVID-19, and we therefore hypothesized that there may be a direct interaction between SARS-CoV-2 and the NCS [10].

Considering the above, we focused on the potential interaction between SARS-CoV-2 and the nicotinic cholinergic system, particularly α7 nAChRs [10]. Any such interaction could have potential therapeutic implications considering that there are pharmacological agents, such as nicotine, which could protect these receptors through competitive binding to the receptors. The identification of a potential interaction between any virus protein and the cholinergic pathway could promote a better understanding of the large spectrum of COVID-19 clinical manifestations and could lead to the identification of potentially therapeutic compounds among the many cholinergic agonists. Additionally, such interactions could explain the previously mentioned clinical observations of under-representation of smokers among hospitalized COVID-19 patients. This would establish the hypothesis that SARS-CoV-2 disrupts the cholinergic anti-inflammatory pathway and causes a variety of clinical manifestations by interacting with nAChRs, and nicotine could prevent this by restoring the function of these receptors.

While tobacco cigarette smoke contains thousands of compounds, many of which are highly toxic and are associated with combustion, we focused on nicotine which has well-established pharmacological effects and has been available for years as an approved medication in various forms. Therefore, nicotine represented a compound with promising prospects if proven to be associated with therapeutic benefits, while additional approved compounds act on nicotinic cholinergic receptors and could also be examined for potential effects.

In this study, we compared amino acid sequences between SARS-CoV-2 and snake venom neurotoxins. The latter are well-established inhibitors of the NCS [16,17]. Subsequently, molecular modelling and docking experiments were performed to examine if there may be an interaction between SARS-CoV-2 Spike Glycoprotein and nAChRs. Based on these findings, we also present the hypothesis that nicotine and other nicotinic cholinergic agonists may have a therapeutic value in COVID-19.

## 2. Results

### 2.1. Sequence Alignment

**Figure 1:**
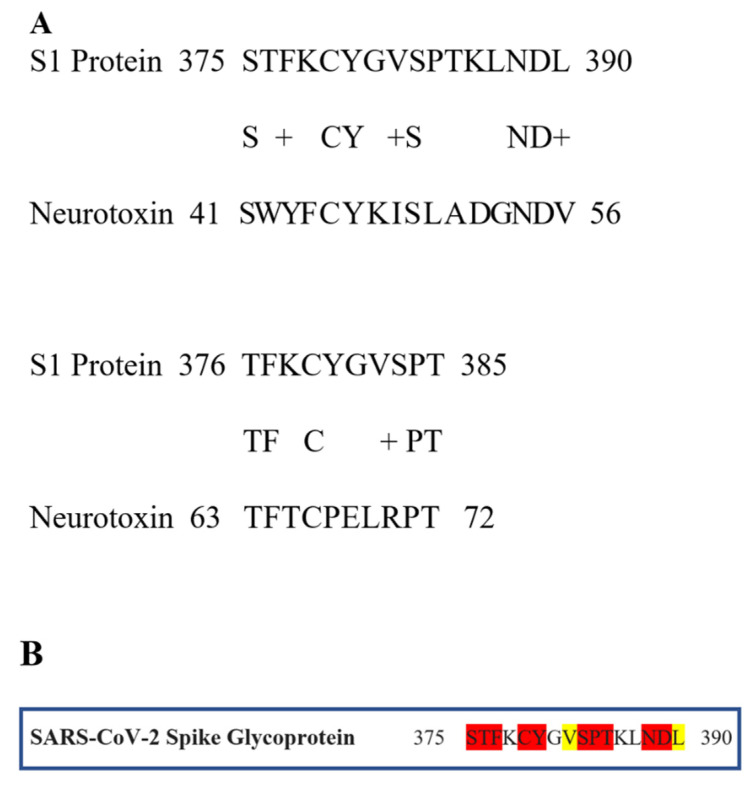
(**A**). Sequence alignment between the SARS-CoV-2 Spike (S1) Glycoprotein (P0DTC2—upper lines) and Neurotoxin homolog NL1 (Q9DEQ3—lower lines). (**B**) Amino acids, within this sequence, which are identical (red) or functionally equivalent (yellow) to Neurotoxin homolog NL1 toxin are shown.

**Figure 2:**
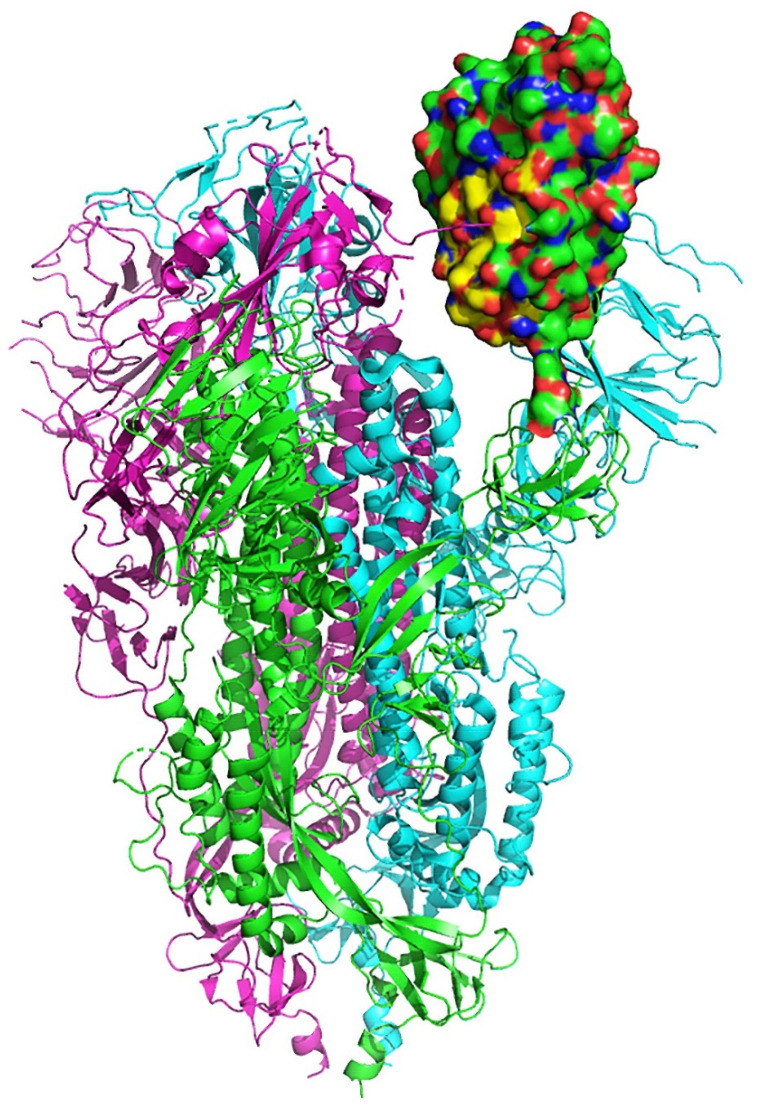
Structural location of the toxin-like sequence (aa 375–390) within the SARS-CoV-2 Spike Glycoprotein. Receptor Binding Domain is in green and the aa 375–390 peptide is in yellow.

Figure 1A presents the sequence alignment between the SARS-CoV-2 Spike Glycoprotein (P0DTC2—upper lines) and Neurotoxin homolog NL1 (Q9DEQ3—lower lines). We identified a double “recombination” within the same sequence of S protein (aa 375–390) which is homologous in sequence to an equivalent of the neurotoxin homolog NL1, part of the toxin’s “three-finger” interacting motif. Figure 1B presents the amino acids within this sequence which are identical (red) or functionally equivalent (conservative replacement, having similar biochemical properties—yellow) to Neurotoxin homolog NL1 toxin. This peptide fragment (aa 375–390) is part of the Receptor Binding Domain (aa 319–541) of the SARS-COV-2 Spike Glycoprotein (the domain through which the Spike protein recognizes the ACE2 on the host’s cell surface) neighboring to the ACE2 Receptor Binding Motif (aa 437–508). Figure 2 displays the structural location of the toxin-like sequence (aa 375–390) within the SARS-CoV-2 Spike Glycoprotein. The sequence is located in the Receptor Binding Domain (next to the Receptor Binding Motif) of the SARS-CoV-2 Spike Glycoprotein.

### 2.2. Interaction between SARS-CoV-2 and nAChRs

**Figure 3:**
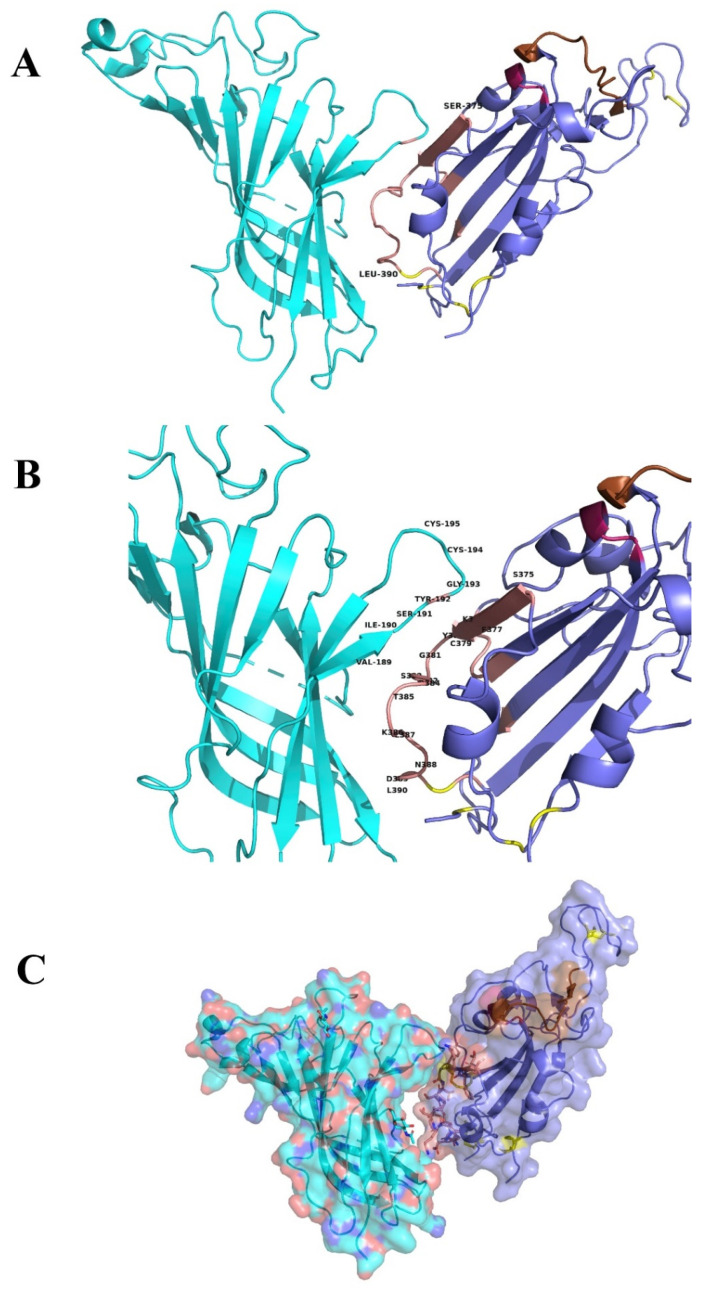
(**A**) Molecular docking of SARS-CoV-2 Spike Glycoprotein (purple) with α9 AChR extracellular subunit (green). The interaction between the two proteins is caused by hydrogen bonds and shape complementarity. Spike glycoprotein is interacting with the toxin-like sequence (aa 375–390) (brown color). (**B**) The interaction between α9 nAChR subunit (aa 189–195 are forming the “toxin binding site’) and the binding site with more details. (**C**) Surface models.

**Figure 4:**
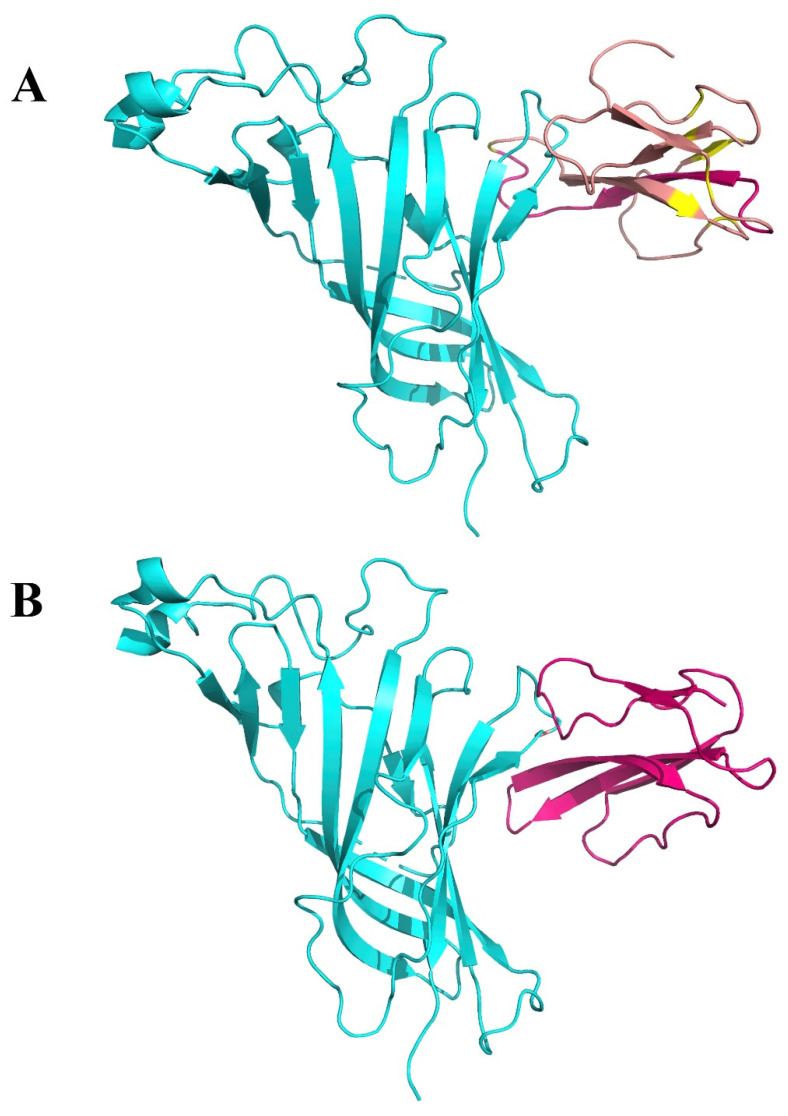
(**A**). Interaction between α9 AChR (green—on the left) and α-bungarotoxin (brown—on the right). (**B**). Interaction between α9 AChR (green—on the left) and neurotoxin homolog NL1 (purple—on the right).

**Figure 5:**
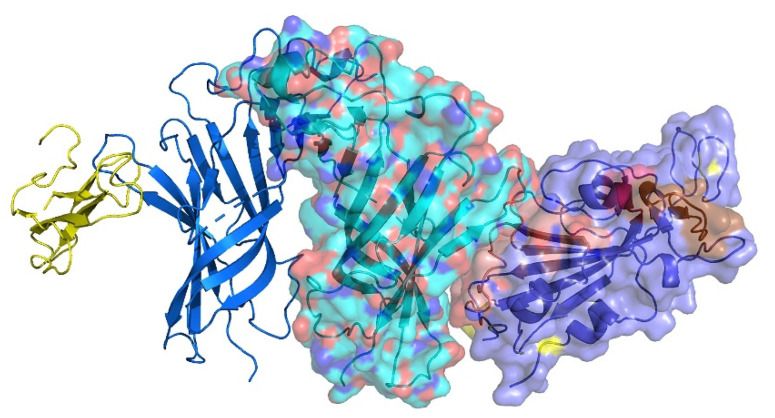
Interaction between two (of the five) α9 nAChR subunits (green—in the middle), alpha-bungarotoxin (yellow—on the left) and SARS-CoV-2 Spike Glycoprotein (purple—on the right).

**Figure 6:**
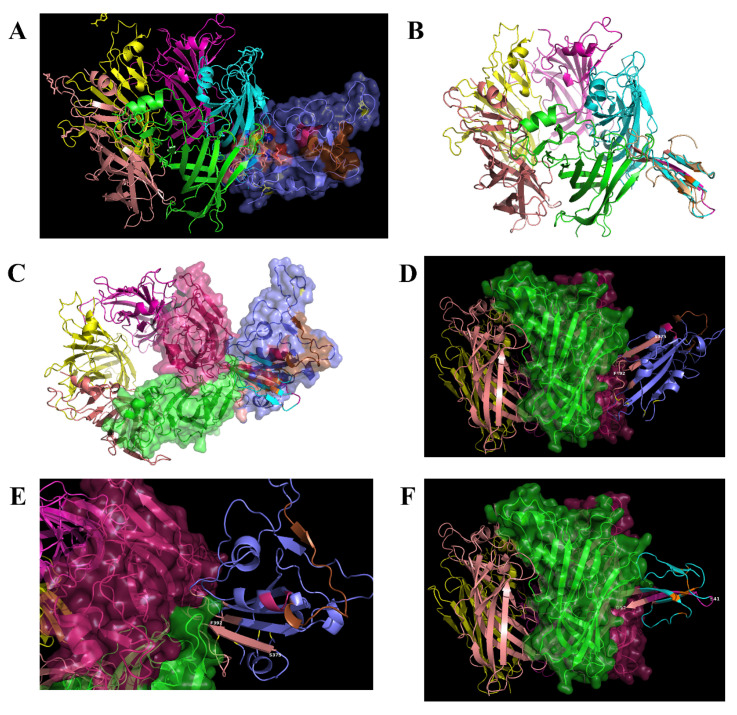
(**A**) Interaction between the ligand binding domain of a pentameric α7 nicotinic receptor chimera (left) and SARS-CoV-2 Spike Glycoprotein (purple—on the right). (**B**) α-bungarotoxin and Neurotoxin homolog NL1 bound to the pentameric nicotinic receptor. (**C**) SARS-CoV-2 Spike Glycoprotein (purple—on the right) bound to the pentameric nicotinic receptor (top view) and side view. (**D**) The beginning of the toxin-like sequence (S375) and the top of the finger-like fragment (F392) are shown. (**E**) Close view of the interacting surfaces. (**F**) Neurotoxin homolog NL1 bound to the pentameric nicotinic receptor in close view, emphasizing the interaction between the toxin and the nicotinic receptor.

The proposed interface region (Figure 3) is formed between the aa 381–386 of the SARS-CoV-2 Spike Glycoprotein and the aa 189–192 of the extracellular domain of the nAChR α9 subunit, a region which forms the core of the “toxin-binding site” of the nAChRs [18]. The interaction between the two proteins is caused by hydrogen bonds and shape complementarity. The mode of interaction is very similar with the interaction between α9 nAChR and α-bungarotoxin and neurotoxin homolog NL1 (Figure 4 and Figure 5), two snake venom toxins which are known to inhibit nAChRs. A similar interaction was found between the ligand binding domain of the pentameric α7 nicotinic receptor chimera and the SARS-CoV-2 Spike Glycoprotein (Figure 6).

### 2.3. Potential Therapeutic Targeting Implications for COVID-19

The in silico experiments of this study identified a potential interaction between SARS-CoV-2 and nAChRs. If verified in vivo and in vitro, these findings could have significant implications in understanding the pathophysiology of COVID-19 and its clinical manifestations, and could also result in novel therapeutic interventions. The hallmark of severe COVID-19 is cytokine storm. This represents a form of immune dysregulation and also a failure of the inflammatory response to return to homeostasis [18]. While the initial response is important in controlling inflammation and infection, uncontrolled cytokine release can be detrimental, leading to tissue damage including acute lung injury and ARDS [19,20]. Case series of COVID-19 patients have shown that elevated levels of cytokines and inflammatory markers predicted adverse outcomes such as mechanical ventilation and death [21,22,23]. Autopsy findings also suggested that severe COVID-19 is associated with immune dysregulation [24].

The nicotinic cholinergic system has been identified as an important modulator of the inflammatory response. The “cholinergic anti-inflammatory pathway” represents a neural pathway of immune homeostasis and cytokine synthesis control [25]. This is a reflex, bi-directional interaction between the nervous and immune system. Sensory input about the inflammatory status transmits information to the central nervous system through the afferent fibers of the vagus nerve, while appropriate responses generate from the efferent fibers to subsequently modulate the inflammatory response and cytokine release [12,26]. This is a rapid reflex mechanism. In 2000, Borovikova et al. first identified that vagus nerve stimulation attenuated the inflammatory response to endotoxin and reduced the production of TNF-α in a rat model of septic shock [27]. Surgical vagotomy significantly enhanced TNF-α response to inflammatory stimuli [12]. Subsequently, several experimental studies identified the α7 nAChR as a key mediator of the neural modulation of inflammation. For example, Wang et al., reported that α7 nAChR knockout mice showed elevated TNF-α production in response to endotoxin, while electrical vagus stimulation did not attenuate this response [13]. Alpha7 nAChRs are present in macrophages and B- and T-lymphocytes [28,29]. Additionally, they are present in human bronchial epithelial cells [16], alveolar epithelial type II cells [30], endothelial cells [15] and neutrophils [31].

The cholinergic anti-inflammatory pathway is particularly active in the lungs. Parasympathetic innervation exists in the airway wall and stimuli are transmitted to the central nervous system through afferent vagus fibers. Pulmonary nociceptors (chemosensitive receptors) were found to be activated by pro-inflammatory cytokines and transmit signals through afferent vagus fibers to the central nervous system [30,32]. Postganglionic cholinergic neurons, which innervate lung tissue, are stimulated through the activation of the efferent vagus nerve fibers, resulting in the activation of α7 nAChR on infiltrated inflammatory cells during acute lung injury, suppressing the production of pro-inflammatory cytokines and attenuating lung injury [33,34]. 

This study identified that there may be a direct interaction between SARS-CoV-2 and nAChRs. This could potentially result in the dysfunction of these receptors and disruption of the cholinergic anti-inflammatory pathway. Nicotinic agonists could protect from this disruption by activating nAChRs. Experimental data have shown that several α7 nAChR agonists, including nicotine, decreased inflammatory manifestations in an acid-induced acute lung injury mouse model [31]. A 60% reduction in excess lung water and extravascular plasma equivalents was observed in nicotine-treated compared to saline-treated groups, while histological examination revealed less pulmonary edema and neutrophil infiltration in the nicotine-treated group. The effects were counteracted by an α7 nAChR antagonist [31]. Additionally, protein concentration, neutrophil counts, cytokine levels and epithelial cell injury were reduced in bronchoalveolar lavage of nicotine-treated mice. In another study examining a model of acute lung inflammatory injury induced by Gram-negative sepsis, nicotine promoted the local suppression of inflammatory mediator production by regulating pro-inflammatory cell transmigration and trans-alveolar permeability [32]. Activation of nicotine also increased survival of mice with Gram-negative pneumonia [32]. Similar protective effects were observed in lipopolysaccharide-induced acute lung injury, with nicotine suppressing the release of several pro-inflammatory cytokines [32,35,36]. These effects appear to be mediated through inhibition of NF-κB activity [31,37,38,39].

Nicotine is an approved medication that has been available for years in different forms, specifically transdermal patches, gums, nasal sprays and oral inhalers and sublingual tablets/lozenges. These formulations are mainly used as smoking cessation interventions and are generally well tolerated and with minimal side effects [40,41]. While nicotine use is currently confined to smokers, prescribed as a smoking substitute, it has been administered therapeutically for the treatment of neurologic or gastrointestinal disorders in non-smoking patients for several weeks, with minimal side effects [42,43,44,45]. In one study, a daily dose of up to 90mg was administered [42]. No nicotine dependence was reported among non-smokers when nicotine was withdrawn after study completion. Therefore, administering nicotine for few days, as is expected to be the treatment duration, for COVID-19 appears to be feasible even for non-smokers—with specific precautions, such as in patients with recent myocardial injury or unstable coronary artery disease. 

Besides nicotine, other nicotinic agonists may have a therapeutic role by protecting nAChRs and activating the cholinergic anti-inflammatory pathway. Choline, a precursor of acetylcholine, was found to suppress murine endotoxemia and sepsis [31,46]. Galantamine is a central acetylcholinesterase inhibitor approved for the treatment of mild to moderate dementia and Alzheimer’s disease. It is also a positive allosteric ligand of nAChRs^42^ and has been shown to stimulate peripheral α7 nAChRs in an experimental model of colitis showing potent anti-inflammatory effects [47]. Experimental studies have shown that it protects against lipopolysaccharide-induced acute lung injury in rats and acid-induced ARDS in rabbits, suggesting that it acts on the cholinergic anti-inflammatory pathway [48]. Varenicline is an approved smoking cessation medication that exhibits strong α7 nAChR agonist activity [49]. One study showed that it exhibits anti-inflammatory property in the lung tissue of mice, mediated via α7 nAChRs [50]. These medications could be proposed for clinical trials, although nicotine has been tested far more extensively in relation to the cholinergic anti-inflammatory pathway. 

**Figure 7:**
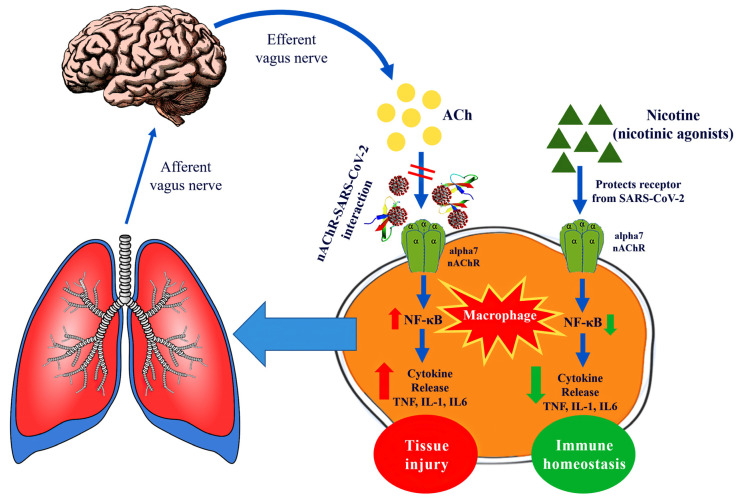
Graphic display of the interaction between SARS-CoV-2 and α7 nicotinic acetylcholine receptors (nAChRs) and the potential therapeutic implications.

An overview of the nicotinic agonists presented above is shown in Table 1. The proposed hypothesis for the interaction between SARS-CoV-2 and nAChRs and the therapeutic implications are displayed in Figure 7.

## 3. Discussion

Animal venoms and especially snake venoms have evolved to contain a wide diversity of proteins that induce inflammatory and toxic effects [17]. Their pharmacological properties have been well-studied, revealing a complex mode of action. Many of these toxins exert their action by binding to the muscle or the neuronal type nAChRs [16,20]. Neurotoxins, such as α-bungarotoxin, interact with the ACh binding site of nAChRs with the sequence aa185–200 being of great importance for such binding [51,52].The molecular modelling and docking experiments presented in this study suggest an interaction between nAChRs and SARS-CoV-2 Spike Glycoprotein, with the sequence aa189–195 of the nAChR being at the core of this interaction. This could compromise the NCS and the cholinergic anti-inflammatory pathway, leading to a hyper-immune response and cytokine storm. 

The consistent observations of a low rate of smoking among hospitalized COVID-19 patients (despite the limitations and perplexities), the potential links between dysfunction of the NCS and clinical manifestations of COVID-19 and the indications for a direct interaction between SARS-CoV-2 and nAChRs leading to NCS dysregulation generate the hypothesis for a novel therapeutic intervention aiming at restoring the function of the cholinergic anti-inflammatory pathway and promoting immune homeostasis [10]. Therapeutic interventions to reduce the hyper-immune response have already been suggested and are currently underway, in some cases for medications with warnings and precautions for their use in active infections [53,54,55,56,57]. While our hypothesis is similarly oriented to controlling the cytokine storm, a different pathway is proposed with medications that are relatively safe and not contraindicated for use in active infections. Furthermore, it should be clarified that the interaction and potential implications presented in this study are not linked to the renin-angiotensin system which, through ACE2, is involved in viral cell entry and replication. We postulate that the pathophysiological mechanisms through which the virus causes severe disease, relevant to an uncontrolled response of the immune system to viral invasion and failure to return to homeostasis, are at least partly different from the mode of cell entry and replication. The findings presented herein suggest that a different pathway may be targeted as a mediator for COVID-19 progression and associated symptoms, the nicotinic cholinergic system. We also provide insight about the potential therapeutic role of already approved medications, which can be used through repurposing, in alleviating symptoms and preventing disease progression without hindering viral replication.

A limitation of this study is that it is based on a theoretical model, and there is currently no in vitro or in vivo study that has examined the possibility of an interaction between SARS-Cov-2 and nAChRs. The study was initiated based on clinical observations about the association between smoking and COVID-19 among hospitalized patients. Tobacco cigarette smoke contains thousands of compounds, most of which have known toxic effects. The possibility that other chemicals besides nicotine may be associated with a potentially beneficial effect in COVID-19 cannot be excluded. For example, low levels of carbon monoxide may have anti-inflammatory properties and have been used experimentally for lung sepsis [58]. The results of this in silico study suggest a plausible mechanism through which nicotine may be implicated in the course of COVID-19 but does not examine the effects of other chemicals present in tobacco cigarette smoke. 

The activation of nAChRs by either endogenous (acetylcholine) or exogenous agonists is induced by opening the ion channel in the receptor, allowing the flow of cations, and results in a variety of biological responses. nAChR antagonists, such as α-neurotoxins, compete with typical agonists for binding, and their binding is restricted to nAChR α-subunits. Nicotine and other nicotinic cholinergic agonists (choline, varenicline and galantamine) are FDAapproved drugs for a number of pathologies (including for smoking cessation) and may reverse this binding, by competing for binding with the SARS-CoV-2 Spike Glycoprotein, and promote the activity of the cholinergic anti-inflammatory pathway. This needs to be studied further and eventually verified in in vitro and in vivo studies.

## 4. Materials and Methods

### 4.1. Sequence Retrieval and Alignment

To examine this hypothesis, we compared amino acid sequences between SARS-CoV-2 and snake venom neurotoxins. The latter are well-established inhibitors of the NCS [17]. The protein sequences of the “three finger” neurotoxin and the SARS-CoV-2 Spike Glycoprotein were retrieved from the National Center for Biotechnology Information (NCBI, Bethesda, MD, USA) database with details (designation and accession numbers) listed in Figure 1. Mega BLAST [59] was used for Blastp (protein-protein BLASTS) searches at the UNIPROT database (and PDB and SwissProt). ClustalOmega (Clustal-O) [60] was used to perform all the multiple sequence alignment programs. Default parameters were used for the alignment.

### 4.2. Structure Retrieval, Alignment and Modelling

The three dimensional structures of the SARS-CoV-2 Spike Glycoprotein (S1) in complex with the human angiotensin converting enzyme 2 (hACE2) (PDB id: 6LZG), the hACE2 (1R41, 1R42), the cryo-EM determined complex of spike protein S-ACE2-B^0^AT1 neutral amino acid transporter (PDB id: 6M18), the structure of a neutralizing to SARS-CoV monoclonal antibody that also cross reacts with the S protein of SARS-CoV-2 when the latter is in complex with the ACE2 receptor (PDB id: 6NB7) and the extracellular domain of the nAChR α9 subunit in complex with α-bungarotoxin (PDB id: 3U8M) were downloaded from the Protein Data Bank [61] and used to analyze the consequences to structure and function of the interaction between S1 and nAChR. The crystal structures of the ligand-binding domain of a pentameric α7 nicotinic receptor chimera (PDB id: 3SQ9) were used for additional structural comparisons [62]. All figures depicting 3D models were created using the molecular graphics program PyMOL V.2.2 [63]. Molecular modelling and molecular docking studies were performed using the software packages MOE and PyMol [63,64]. Since the structure of the human α7 nAChR is not solved yet, we used the crystal structure of the extracellular domain of the homologous α9 nAChR bound to α-bungarotoxin.

## 5. Conclusions

This in silico study identified a potential interaction between SARS-CoV-2 and nAChRs, which could result in dysregulation of the cholinergic anti-inflammatory pathway and could adversely affect immune homeostasis in COVID-19. These findings have potential therapeutic implications since cholinergic agonists could protect and restore the function of nAChRs. Nicotine, varenicline, and galantamine—examples of FDA-approved cholinergic agonists for various pathologies—are proposed as therapeutic interventions for COVID-19, targeting the NCS. Since these proposals are based on theoretical models, further in vitro and in vivo studies are needed to explore these pathophysiological mechanisms.

## Figures and Tables

**Table 1:** Approved nicotinic cholinergic agonists that could be tested in in vitro and in vivo studies for their effects on COVID-19.

Compound	Mode of Action	FDA Approval	Approved Indication	Dose	Brand Name
Choline	Acetylcholine synthesis precursor	(1)	Dietary supplement	550 mg/day (men) 425 mg/day (women) (2)	Various
Nicotine	Alpha7 AChR agonist	Yes	Smoking cessation	Maximum dose (3) 21 mg/day (patch) 40 mg/day (nasal spray) 64 mg/day (inhaler) 96 mg/day (gum)	Nicorette Nicoderm Nicotrol Others
Galantamine	Weak acetylcholinesterase inhibitor Allosteric agonist for nicotinic acetylcholine receptors	Yes	Alzheimer’s disease	16–24 mg/day	Reminyl Razadyne
Varenicline	Alpha7 AChR agonist Alpha4 beta2 AChR partial agonist	Yes	Smoking cessation	2 mg/day	Champix Chantix

(1) Choline is an essential nutrient and is available as a dietary supplement in various formulations. (2) The dose refers to Adequate Intake (AI). (3) Nicotine dose refers to smokers who are nicotine users (through smoking) and, thus, have developed tolerance.
